# Electrospun cellulose acetate doped with astaxanthin derivatives from *Haematococcus pluvialis* for *in vivo* anti-aging activity†

**DOI:** 10.1039/c8ra08156e

**Published:** 2018-11-05

**Authors:** Jukkrit Nootem, Pawanrat Chalorak, Krai Meemon, Withawat Mingvanish, Kornkanya Pratumyot, Leela Ruckthong, Choladda Srisuwannaket, Nakorn Niamnont

**Affiliations:** Organic Synthesis, Electrochemistry & Natural Product Research Unit, Department of Chemistry, Faculty of Science, King Mongkut's University of Technology Thonburi Bangkok 10140 Thailand nakorn.nia@kmutt.ac.th; Department of Anatomy, Faculty of Science, Mahidol University Bangkok 10400 Thailand

## Abstract

This research aims to study the release, *in vivo* anti-aging activity against *Caenorhabditis elegans* and stability of astaxanthins in a crude acetone extract of *Haematococcus pluvialis* from electrospun cellulose acetate (CA) nanofibers. The content and 2,2-diphenyl-1-picryl-hydrazyl-hydrate (DPPH) radical scavenging activity of astaxanthins in the crude extract were also determined. The content of astaxanthins was reported in terms of total carotenoid content (TCC) and found to be 10.75 ± 0.16 mg gcE^−1^. IC_50_ of DPPH radical scavenging activity for astaxanthins was 233.33 ± 4.18 μg mL^−1^. It has been well known that astaxanthins are very unstable under environmental conditions, so the electrospinning technique was used to enhance their stability. In order to fabricate CA nanofibers containing a crude acetone extract of *H. pluvialis*, various solvent systems and percent loading of the crude acetone extract were studied. The optimal solvent system for fabrication of CA nanofibers was the acetone/dimethylformamide (DMF) system (2 : 1 v/v) with incorporation of 0.25% v/v Tween80, resulting in good morphology of CA nanofibers with av. 420 nm diameter. The loading efficiency (%) of the crude astaxanthins extract was 5% w/w of CA. With regard to the results of the *in vivo* oxidative stress assay, *C. elegans* pre-treated with 200 μg mL^−1^ of the crude extract had a survival percent of 56 after administration of 250 mM of paraquat for 8 h. Under phosphate-buffered saline (pH 7.4) containing 10% v/v acetone, the release of astaxanthins from the CA nanofibers loaded with the crude extract exhibited a prolonged profile. The stability of astaxanthins in electrospun CA nanofibers was examined using the freeze–thaw cycle testing through a DPPH radical scavenging assay. It was found that their stability was significantly different (*P* < 0.05) after the 12^th^ freeze–thaw cycle compared with the crude extract.

## Abstract 2

The astaxanthin derivatives loaded CA fibers enhanced the percentage survival of *C. elegans* upto 56.17% after adding paraquat and it stability increased with 1.7 times by using DPPH scavenging assay.

## Graphical abstract

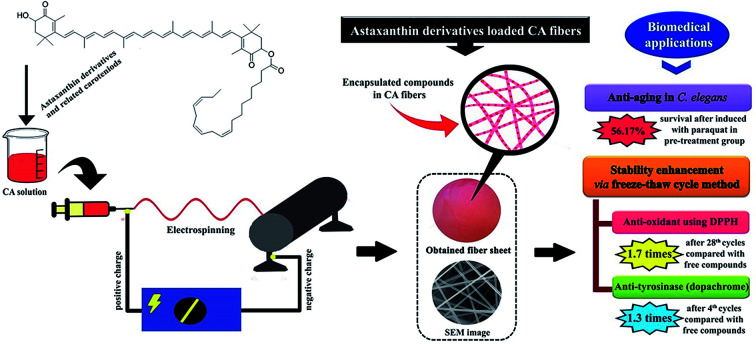

## Introduction

Astaxanthin and its esters are naturally occurring and blood-red carotenoid pigments. They have been found in rainwater microalgae (*Haematococcus pluvialis*), the yeast fungus called *Xanthophyllomyces dendrorhous* (also known as *Phaffia*), Antarctic krill, marine copepods, deep sea shrimp, and edible crab shells.^1,2^ However, astaxanthins has been mainly found in *Haematococcus pluvialis*. Astaxanthin is considered as a natural super anti-oxidant as compared with the naturally occurring carotenoids.^3^ For examples, astaxanthin exhibited 10 times more antioxidant activity than zeaxanthin, lutein, canthaxanthin and β-carotene, and 100 times higher than vitamin E.^4^ Other benefits of astaxanthin in biological activities are anti-inflammation, anti-diabetic activity, anticancer activity, anti-tyrosinase activity, *etc.*^5,6^

Most structures of natural astaxanthin have been found in the form of mono- and diesters; 70% monoesters of astaxanthins, 10% diesters, 5% free astaxanthin and the remaining 15% contains a mixture of β-carotene, canthaxanthin, lutein and other carotenoids. Because of their structure containing high multiple bonds, they are easily oxidized and unstable upon exposure to heat, light and oxygen, which result in the decrease in their biological activities.^7^ One simple and versatile technique for helping the astaxanthin stability is encapsulation with chitosan, cyclodextrin, calcium alginate, *etc.*^8–10^ However, the encapsulation technique is limited with difficulty of handling, low loading of bioactive compounds and upscaling for mass production.^10^ A new technique for delivery of bioactive compounds is electrospinning technique, which produced continuous nano to submicro fibers using electrostatic forces.^11–13^ The characteristic advantages of encapsulation technique for delivery of bioactive compounds include high drug loading, high encapsulation efficiency, variety of polymers compatible with bioactive compounds, ability to modify drug release rate, process simplicity and cost effectiveness.^14,15^

Furthermore, fibers produced using electrospinning have high surface area-to-volume and aspect ratios, which can promote the release of bioactive compounds.^16,17^ The most widely used matrix polymer for electrospinning technique is cellulose acetate (CA) due to its biodegradable and non-toxic properties.^18^ Since CA is not too hydrophobic and not hydrophilic, CA enables water to permeate into fibers and make hydrophobic bioactive compounds diffuse to human skin or organism. Many researches have been reported on the study of the release of bioactive compounds such as vitamin E, vitamin A, and gallic acid from CA nanofibers. They showed a significant increase in their releasing profile against a media buffer as compared with a casting film.^19,20^

In this work, we have reported our attempt to enhance the stability of astaxanthins extracted from *H. pluvialis* by doping it with CA fibers upon electrospinning technique. The ability of drug carrier has been tested through *in vitro* DPPH radical scavenging activity and *in vivo* oxidative stress assay with *C. elegans*. These results will facilitate the implementation of electrospinning techniques to stabilize astaxanthins and control quantity of astaxanthins released.

## Results and discussion

### Extraction and characterization of astaxanthin derivatives in *H. pluvialis*

Astaxanthin derivatives in *H. pluvialis* were extracted with acetone with its percent yield of 21.50 ± 3.52% (w/w). The crude astaxanthin extract was then purified by column chromatography resulting in 4.22 ± 2.05% (w/w) of astaxanthin derivatives and accounted for 0.91 ± 0.15% (w/w) of dry biomass. ^1^H-MNR and ^13^C-NMR spectroscopic techniques were used to characterize and identify as astaxanthin esters; mainly astaxanthin monoesters of fatty acids (Fig. S1–S3 in ESI†). According to the research work of Lorenz *et al.*,^21^ almost 70% of the astaxanthin derivatives were found to be astaxanthin monoester, while the remaining constituted astaxanthin diester, astaxanthin free, lutein, canthaxanthin and β-carotene. Since the amount of purified astaxanthin monoester was relatively small. So it was used in the form of the crude extract for further experiments.

### Determination of total carotenoid content and DPPH radical scavenging activity of the crude astaxanthin extract from *H. pluvialis*

The secondary metabolites reported to be found in *H. pluvialis* contain chlorophyll *a*, chlorophyll *b*, and carotenoids and their derivatives. The levels of chlorophyll *a*, chlorophyll *b* and total carotenoid contents were determined using UV-Vis spectrophotometer (Table S1†). The amounts of chlorophyll *b* and chlorophyll *a* in the crude extract were 2.54 mg gcE^−1^, and 8.72 mg gcE^−1^, respectively. Total carotenoid content was found to be 10.75 mg gcE^−1^. However, under the proper stress conditions, *Haematococcus* encysts and produces high concentrations of carotenoids, which facilitates its own protection against light and oxygen.^1^ With regarding to DPPH radical scavenging testing, this crude extract gave IC_50_ 233.33 ± 4.18 (μg mL^−1^).

### The effects of solvent systems and Tween80 on the morphology of CA nanofibers

**Fig. 1:**
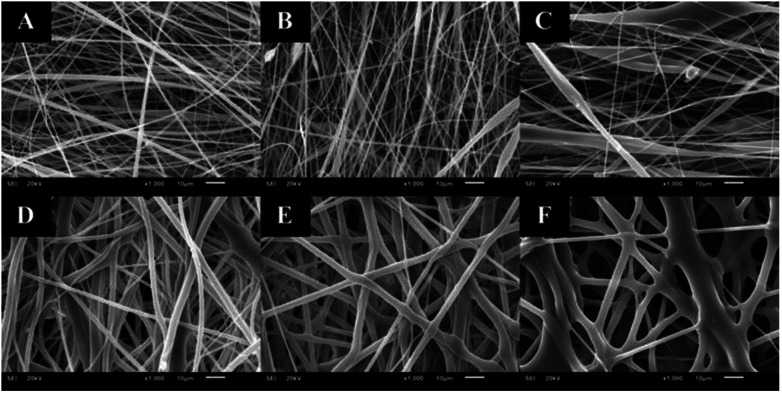
CA electrospun nanofibers from various types of solvent system and volumetric ratios including: (A) acetone/DMF at 2 : 1, (B) acetone/DMF at 3 : 1, (C) acetone/DMF at 4 : 1, (D) EA/DMF at 2 : 1, (E) EA/DMF at 3 : 1, and (F) EA/DMF at 4 : 1, respectively.

**Fig. 2:**
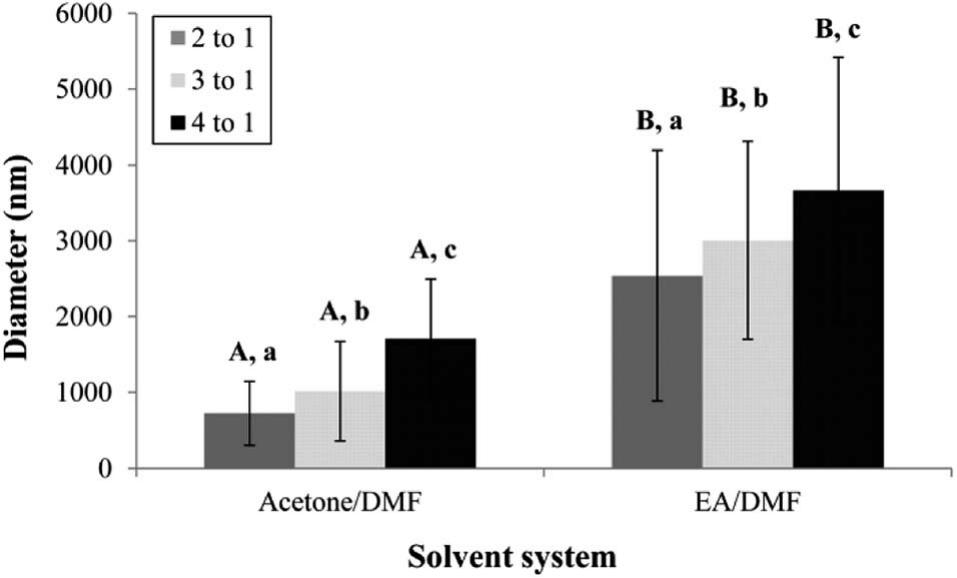
The average diameter of CA electrospun nanofibers from various types of solvent system and volumetric ratios. ^A,B^Means with different capital letter superscripts are significantly different (*P* < 0.05) at the same ratios of solvent.

**Fig. 3:**
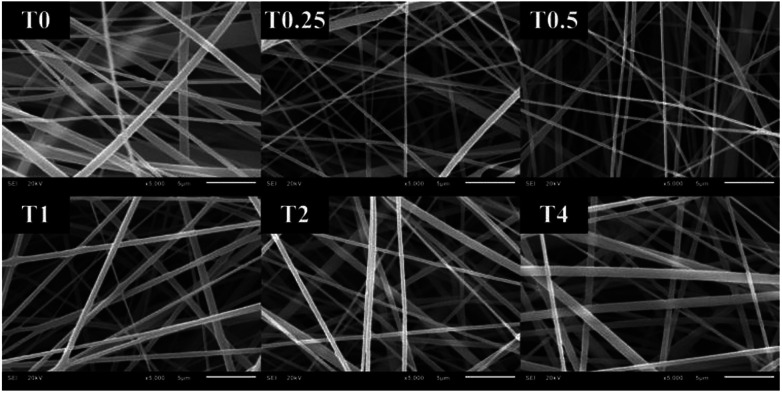
CA electrospun nanofibers from various volumes of Tween80 including: (T0) 0%, (T0.25) 0.25%, (T0.5) 0.5%, (T1) 1%, (T2) 2%, and (T4) 4% v/v, respectively.

**Fig. 4:**
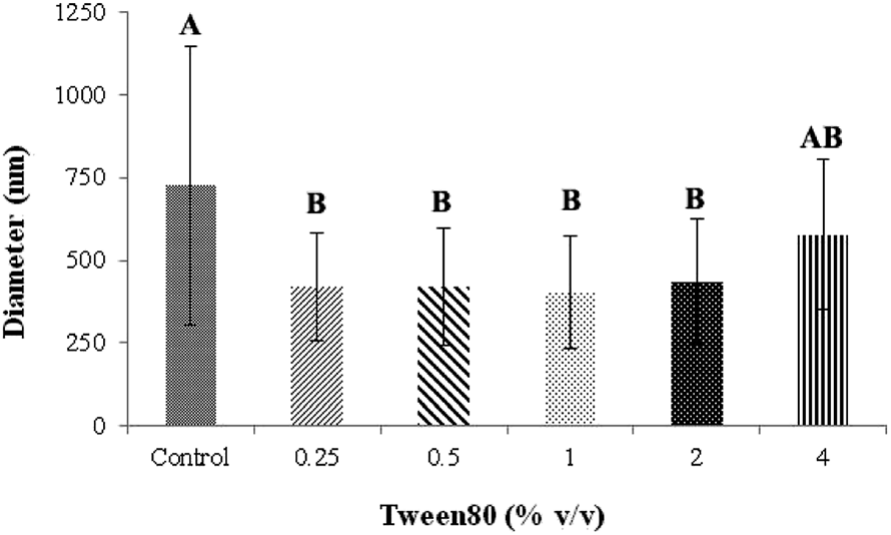
The average diameter of CA electrospun nanofibers from various volume of Tween80. ^A,B^Means with different capital letter superscripts are significantly different (*P* < 0.05).

The distance between the tip and the corrector on fiber morphology is significant. So, the suitable distance was seated at 15 cm.^22^ This research aimed to study the effects of solvent systems and surfactant. The morphology of electrospun nanofibers from various types of solvent systems with different ratios was shown in Fig. 1. The average diameters of fibers obtained from the acetone/DMF system with the volume ratios of 2 : 1, 3 : 1 and 4 : 1 were 725 ± 419, 1016 ± 653 and 1709 ± 787 nm, respectively; whereas the relative ratios appeared to be 2538 ± 1654, 3006 ± 1305 and 3668 ± 1751 nm in the EA/DMF system (Fig. 2). Obviously, the different solvent systems dramatically affected the morphology of CA nanofibers due to their different polarity, conductivity, and evaporation rate.^14^ In terms of solvent types, acetone has higher polarity and evaporation rate than EA, thus leading to the different formations of solid-state characteristics of CA nanofibers. At the same ratios of the solvent systems, the average diameters of CA nanofibers were significantly different at *P* < 0.05. The acetone/DMF system possessed a proper evaporation rate, subsequently making the CA nanofibers smaller in diameters, while the EA/DMF system was hard to vaporize and revealed non-uniform and larger diameters.^23^ Regardless of solvent systems, an increasing ratio of acetone and EA influenced the morphology of fibers which significantly affected their diameters (*P* < 0.05). Rapid evaporation of solvents caused in the coagulation of solid-state at the needle tip prior to elongation and resulted in a broad diameter distribution. Additionally, a co-solvent system with an acetone/DMF ratio of 4 : 1 demonstrated high amount of beads compared to the EA/DMF system at the same ratio. The best characteristic of fibers obtained from the acetone/DMF system at a ratio of 2 : 1 was therefore collected to study the effect of Tween80 surfactant on its morphology. Since Tween80 is non-ionic surfactant, it could not interrupt with the electrospinning process compared with other cation or anionic surfactants.^24^ Moreover, Tween80 also exhibits low toxicity and causes less irritation to human skin, which makes it suitable for pharmaceutical and cosmetic applications.^25^ The addition of Tween80 at 0.25, 0.5, 1 and 2% v/v to a solution of CA decreased fibers' average diameter and reduced the standard deviation (Fig. 3 and 4) to be 420 ± 163, 419 ± 198, 405 ± 170 and 434 ± 203 nm, respectively as compared with the average diameter of the surfactant free CA nanofibers (725 ± 419 nm). The average diameter of nanofibers containing Tween80 was significantly different (*P* < 0.05) from those without Tween80 incorporation.^26^ When increasing Tween80 to a proper volume, the surface tension still decreased and the average diameter of fibers became smaller, while excess volume at 4% v/v of Tween80 resulted in a slight increase in fiber average diameter. Tween80 is a surfactant with high molecular weight and high level of volatility. It could therefore affect the solvent by increasing the evaporation rate of the polymer solution. The obtained result is consistent with the research work of Jia *et al.*,^27^ in which it was stated that the exceed amount of non-ionic surfactant caused formation of free micelles that consequently disturbs the electrospinning process. As a consequence, relatively larger average diameter of nanofiber was generated. However, the average diameters of CA nanofibers obtained from 4% v/v Tween80 addition appeared to be not significantly different from those obtained from the other volume ratios (0.25–2%). Because of no significant difference in nanofiber's average diameters among the variation in Tween 80 content, the lowest amount of Tween80 at 0.25% v/v was collected to study in subsequent processes.

### Percent loading (actual drug content)

**Fig. 5:**
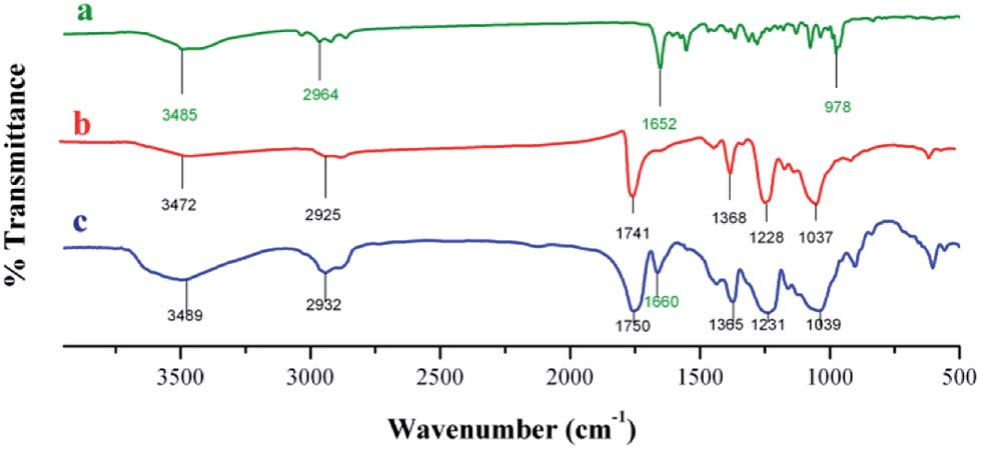
IR spectra of (a) crude astaxanthin extract, (b) CA electrospun nanofibers, and (c) crude astaxanthin extract loaded CA electrospun nanofibers.

Crude astaxanthin extract-loaded CA nanofiber mats were used to investigate the actual drug content after electrospinning procedure. Optimally, the crude astaxanthin extract was dissolved with 17% w/v of CA in acetone/DMF (2 : 1 ratio) containing 0.25% v/v of Tween80 to give good morphology of fibers. The crude astaxanthin was obtained in 76.79 ± 4.98% (compared to the initial amount of crude astaxanthin in the polymer solution). The loss of drug content during electrospinning was caused from high electrical voltage, oxygen, and heat accumulation at the spinning collector. With all these factors altogether, some of the unsaturated backbone of carotenoid compounds can be oxidized. FT-IR spectra of astaxanthin derivatives (Fig. 5 and Table S2†) illustrated a specific peak at 1652 cm^−1^, which corresponds to a C=C bond. Moreover, there is an additional peak at around 1660 cm^−1^ which is the characteristics of astaxanthin derivatives-loaded CA nanofibers. This showed that the unsaturated C=C bond of carotenoid compounds remained stable after fabricating by electrospinning method.

### Effect of percent loading on anti-oxidant activity

**Fig. 6:**
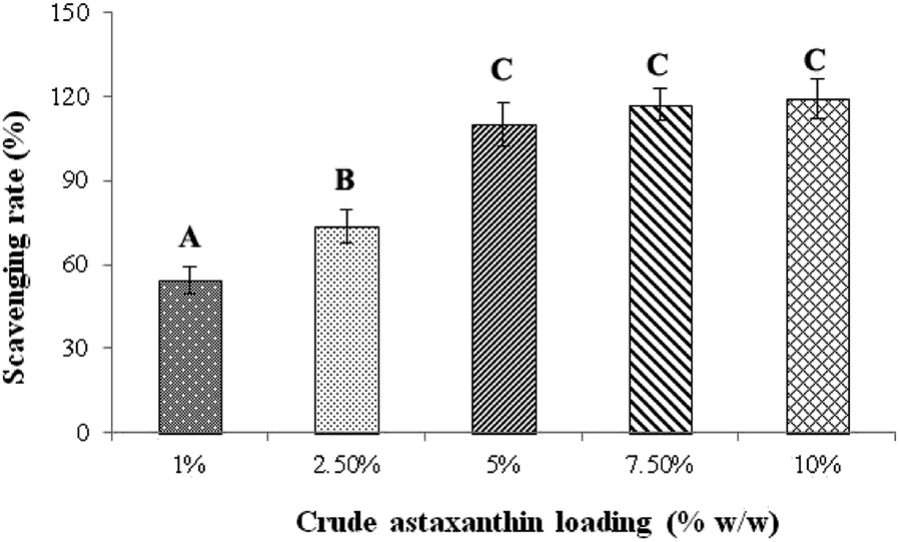
Effect of percentage loading of crude astaxanthin extract on scavenging rates. ^A,B,C^Means with different capital letter superscripts are significantly different (*P* < 0.05).

The effect of percent loading of astaxanthins in CA nanofiber fabricated from 17% w/v CA in acetone/DMF (2 : 1 ratio) and 0.25% v/v of Tween80 was studied by following the DPPH radical scavenging activity. Fig. 6 is the plot of percent loading of astaxanthins against DPPH scavenging rate. The percent loading at 1, 2.5, 5, 7.5 and 10% w/w yielded the DPPH scavenging rates of 54.2 ± 4.81, 73.5 ± 5.84, 110 ± 7.72, 117 ± 5.63 and 119 ± 7.25% w/w, respectively. However, the DPPH scavenging rates at the percent loading of 5, 7.5 and 10% w/w were not significantly different (*P* < 0.05). Thus, the lowest percent loading with the highest DPPH scavenging rate of 5% w/w was selected to study in further experiments to save cost of studies and prevent the waste of excess amount of astaxanthins.

### Crude astaxanthin renders *C. elegans* resistant to oxidative stress

**Fig. 7:**
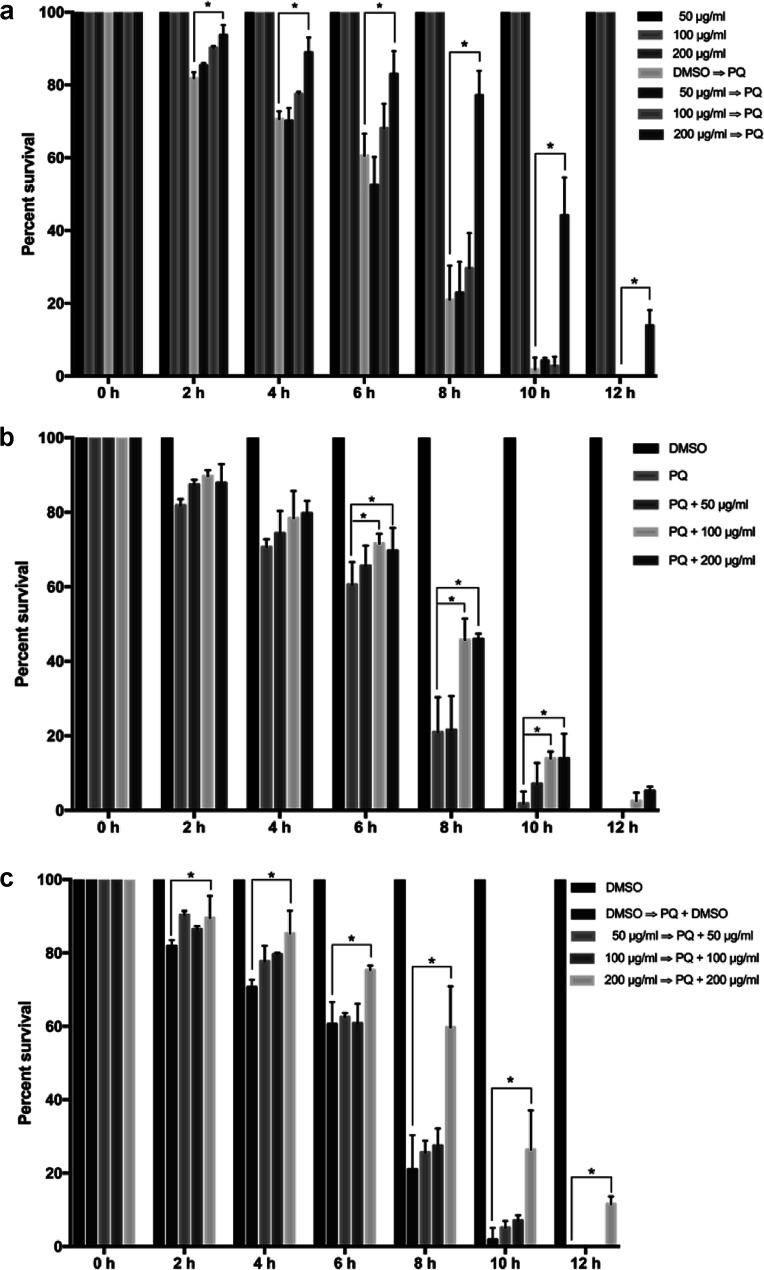
Oxidative stress resistance of crude astaxanthins at various concentrations in *C. elegans* for (a) pre-treatment group, (b) co-treatment group, (c) all-treatment group, respectively.

To evaluate the astaxanthins' capacity against oxidative stress of *C. elegans*, the oxidative stress assay was performed under three conditions: pre-treatment with the crude astaxanthin extract, co-treatment with both the crude astaxanthin extract and 250 mM paraquat, and all-treatment. Compared to the oxidative stress resistance of the control group the pre-treated worms with 200 μg mL^−1^ of the crude astaxanthin extract had the significantly increasing survival percentage after being induced with 250 mM paraquat. It was found that the survival percentages were 11.78%, 18.21%, 22.45%, 56.17%, 42.30% and 13.88% at 2, 4, 6, 8 and 12 h, respectively, (*p* < 0.05). However, the pre-treatment with 50 and 100 μg mL^−1^ of the crude astaxanthin extract did not significantly increase the survivability. The controlled worms that were only treated with various concentrations of the crude astaxanthin extract could survive for 12 h, which indicates the non-toxicity of the crude astaxanthin extract (Fig. 7a).

### Release of astaxanthins-loaded CA nanofibers

**Fig. 8:**
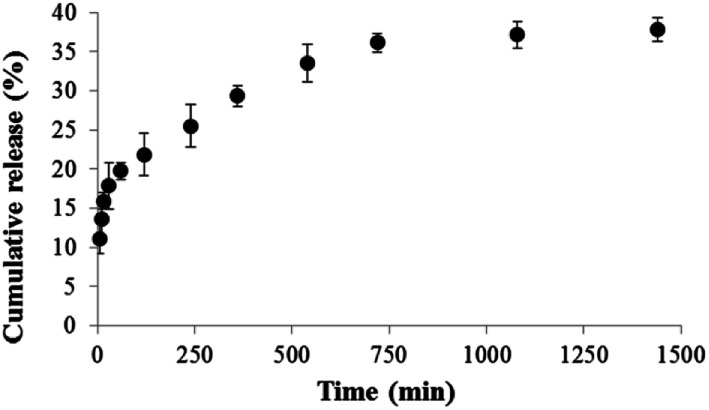
Cumulative astaxanthins release of crude astaxanthin extract loaded CA electrospun nanofibers in PBS containing 10% v/v of acetone and 0.5% w/v of SDS.

The astaxanthins releasing profile of crude astaxanthin extract-loaded CA nanofibers mat is presented in Fig. 8. Increment of time resulted in an increase of astaxanthin release, which exhibited three distinct characteristics. Initially, a burst astaxanthins release of 20% was found within the first 60 min due to a high surface to volume ratio^28^ of CA-spun fiber that enables the nanofiber mat to swell better in the media. Here, some extent of astaxanthins was emitted into the media and formed micelles with SDS. Moreover, some fatty acids such as oleic acid can dissolve well in a media solution containing a small volume of acetone due to their similar polarity. Secondly, in the 60–720 min duration a gradual decrease in astaxanthins releasing rates was due to compound depletion. At this stage, the cumulative astaxanthins release ranged from ∼20–35 in the final interval of the study, the cumulative astaxanthins release was found to be constant. The final cumulative astaxanthins release was 38% at which the rest of the lipophilic compounds could be entrapped within CA nanofibers.^29^ This can be explained by the fact that the interaction between hydrophobic CA polymeric matrix and hydrophobic astaxanthins was greater than interactions between astaxanthins and water molecules in medium. Tsekova *et al.*,^30^ previously reported that the use of mixed spun-fibers of CA/PVP yielded more efficient release of loaded curcumin than that of CA. This is because PVP is the hydrophilic polymer, so it can completely dissolve in media buffer. As a result curcumin-loaded CA/PVP fibers were released effectively.

### Stability of crude astaxanthin extract-loaded CA nanofiber mats

**Fig. 9:**
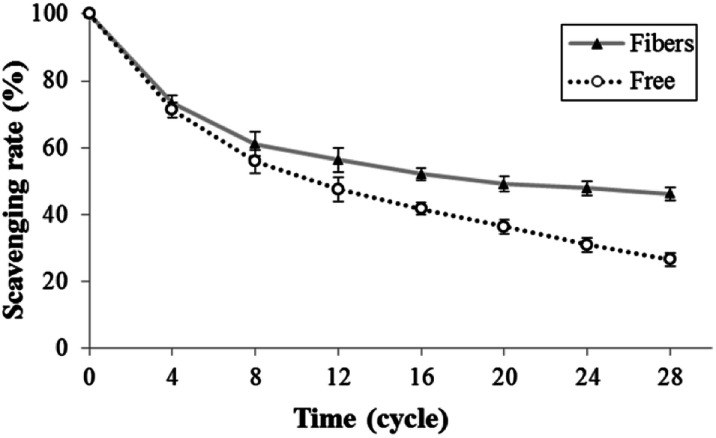
Antioxidant activity *via* the freeze–thaw cycle of (▲) crude astaxanthin extract loaded CA electrospun nanofibers and (○) crude astaxanthin extract.

**Fig. 10:**
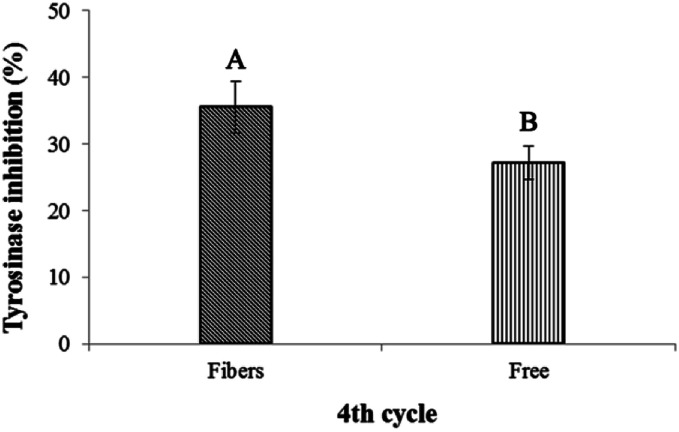
Tyrosinase inhibitory activity *via* the freeze–thaw cycle of crude astaxanthin extract loaded CA electrospun nanofibers and crude astaxanthin extract at 4^th^ cycle. ^A,B^Means with different capital letter superscripts are significantly different (*P* < 0.05).

The stability of astaxanthins-loaded nanofibers was investigated using a freeze–thaw cycle testing. The stability of astaxanthins was determined *via* their remaining DPPH radical scavenging activity as shown in Fig. 9. At the 0^th^–4^th^ cycles, both astaxanthins loaded into CA nanofiber mats and free astaxanthins in crude astaxanthin extract exhibited a rapid fall in DPPH radical scavenging rate upon heating and cooling. A slightly lower DPPH radical scavenging rate of free astaxanthins in crude astaxanthin extract was found at the 8^th^ cycle. The crude astaxanthin extract-loaded CA nanofibers has significantly higher DPPH radical scavenging rate after the 12^th^ cycle as compared with free astaxanthins in crude astaxanthin extract (*P* < 0.05). At the final cycle, it showed an observable difference in their stability with DPPH radical scavenging rate values of 46.18 ± 2.21% for crude astaxanthin extract-loaded CA nanofibers and 26.49 ± 1.86% for free astaxanthins in crude astaxanthin extract, respectively. Regarding to the anti-tyrosinase activity, the percentage of tyrosinase inhibition of crude astaxanthin extract-loaded CA nanofibers (35.56 ± 3.85%) was initially found to be significantly different from that for free astaxanthins in crude astaxanthin extract (27.22 ± 2.55%) at the 4^th^ cycle. The results clearly indicated that CA electrospun nanofibers are a good drug delivery device because it can retain drug stability and at the same time protect the drug from stability testing under simulated conditions (Fig. 10).

## Experimental

### Extraction of astaxanthin derivatives from *H. pluvialis*

The extraction of astaxanthins from *H. pluvialis* was carried out according to the method of Dong *et al.*,^31^ with slightly modified procedure. Briefly, 10 g of dried biomass of *H. pluvialis* was disintegrated in 100 mL of 4 M HCl with stirring at 500 rpm for 5 min. Then, the sediment was washed by 100 mL of distilled water twice and extracted with 100 mL of acetone in ultrasonic bath at 20 °C for 20 min. The acetone-treated solution was evaporated by rotary evaporator under vacuum condition to obtain a crude astaxanthin extract. The crude extract was kept in a round-bottom flask under nitrogen gas at 4 °C.

### Purification and characterization of astaxanthin

The crude astaxanthin extract was further purified by silica gel column chromatography (60–200 mesh) and eluted with a hexane/EA gradient elution (95 : 5, 90 : 10, 85 : 15, 80 : 20, 75 : 25 and 70 : 30 v/v). All fractions were traced with thin layer chromatography (TLC). In order to investigate the presence of astaxanthins in the individual fractions, each fraction was characterized by ^1^H- and ^13^C-NMR techniques using Advanced III HD 400 MHz NMR spectrometer (Switzerland) in chloroform-d.

### Determination of total carotenoid content in crude astaxanthin extract

The solutions of the crude astaxanthin extract at the known concentrations (50–200 μg mL^−1^) were prepared in acetone. The determination of total carotenoid content was carried out by UV-Vis spectrophotometer (Hitachi U-2900, Japan) at 470, 645 and 662 nm following protocols of Lichtenthaler *et al.*^32^ Total carotenoid content, chlorophyll *a* and *b* were calculated according to the equations below.Chlorophyll *a* (*C*_*a*_, mg L^−1^) = 11.75Abs_662_ − 2.35Abs_645_Chlorophyll *b* (*C*_*b*_, mg L^−1^) = 18.61Abs_645_ − 3.96Abs_662_Total carotenoids (mg L^−1^) = (1000Abs_470_ − 2.27*C*_*a*_ − 81.4*C*_*b*_)/227where Abs_470_, Abs_645_ and Abs_662_ are the absorbance of the solutions at 470, 645 and 662 nm, respectively.

### DPPH radical scavenging activity of crude astaxanthin extract

The anti-oxidant activity of the crude astaxanthin extract was measured according to the method of Kirby and Schmidt^33^ with some modifications. 0.1 mM DPPH solution was prepared in 10% v/v acetone in methanol and sample solutions of predetermined concentrations were also prepared 10% v/v acetone in methanol. Each 100 μL of 0.1 mM DPPH solution was added into 100 μL of sample solutions. The mixtures were shook and allowed to stand for 30 min in the dark at room temperature. Then, the absorbance of the mixture was measured by 96-well plate UV spectrophotometer (Perkin-Elmer; EnSpire Multimode Plate Reader, United States) at 517 nm. The scavenging activity was calculated as following equation.
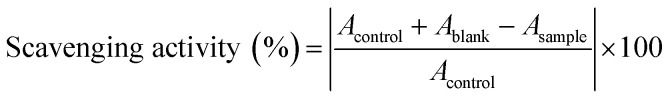
where *A*_control_, *A*_blank_ and *A*_sample_ were the absorbance of 0.1 mM DPPH solution in 10% v/v acetone in methanol, the sample solution without 0.1 mM DPPH solution and the sample solution with 0.1 mM DPPH solution, respectively. The scavenging activity value of the crude astaxanthin extract was reported in terms of half maximal inhibitory concentration (IC_50_).

### Electrospinning and characterization of CA fiber mats

#### Preparation of the CA solutions

17% w/v CA solutions were prepared in various solvent systems containing either acetone/DMF or EA/DMF with the volumetric ratios of 2 : 1, 3 : 1 and 4 : 1. 17% w/v CA solutions were prepared in various solvent systems containing either acetone/DMF or EA/DMF with the volumetric ratios of 2 : 1, 3 : 1 and 4 : 1. Then, each polymer solution of known concentration was loaded in a 5 mL of syringe equipped with a metal needle of an inner diameter of 0.22 mm with the rate of 2 mL h^−1^.

#### Electrospinning condition

For electrospinning, the needle was connected to the positive electrode (Gamma high voltage research FL 32174, United States) and the negative electrode was connected to the rotary spinning collector. The needle tip was located at about 15 cm apart from the collector. Each polymer solution was then injected with applied voltage of 15 kV and the rotary spinning collector was spun at 120 rpm.^34–36^

#### Effect of Tween80 on morphology of CA nanofibers

The optimized conditions for electrospinning CA nanofibers were applied for studying the effect of the concentrations of Tween80 surfactant on its morphology.^37^ Tween80 surfactant was added into the polymer solution at various concentrations (0, 0.25, 0.5, 1, 2 and 4% v/v). In order to examine the morphology of CA nanofiber samples, the samples were dried in a vacuum oven at 50 °C for 3 h. After that, each sample was cut into small pieces (1 × 1 cm) and coated with gold. Then, the morphology of CA nanofibers was observed under a scanning electron microscope (JEOL JSM-6610 LV; SEM, United States). The diameter, diameter distribution and uniforming of each CA nanofiber sample was analyzed using ImageJ 1.51K software (*n* ≥ 100).^38^ For each experiment, the average fiber diameter and distribution were determined from 100 measurements of the random nanofibers. All experiments were conducted at room temperature.

### Studying of crude astaxanthin extract-loaded CA nanofibers

#### Percent loading

The crude astaxanthin extract of a predetermined weight was dissolved in a CA solution of known concentration without adding Tween80 and was electrospun under optimum conditions to afford a CA nanofiber mat. 10 mg of a CA nanofiber mat loaded with the crude astaxanthin extract were re-dissolved in 10 mL of a mixture of acetone and *N*,*N*-dimethylacetamide (2 : 1 v/v). Its solution was examined for the amount of astaxanthins using UV-Vis spectrometer at 475 nm and was reported as mean value + SD (*n* = 3).

#### FT-IR analysis

For qualitative analysis, the CA nanofiber mat loaded with the crude astaxanthin extract (2 cm × 1 cm) was characterized using FT-IR spectrometry (Thermofisher scientific; Nicolet iS5, USA) and its absorption spectra was compared with that without the crude astaxanthin extract. The scanning ranges of spectra were 500–4000 cm^−1^ with a resolution of 16 cm^−1^. Each spectrum was recorded for 32 scans.

#### Effect of percent loading on anti-oxidant activity

In each experiment, a CA solution containing various concentrations of the crude astaxanthin extract (1%, 2.5%, 5%, 7.5% and 10% w/w of CA powder) was homogenized for 5 min in sonication machine and was examined for the DPPH radical scavenging activity. Briefly, 50 mg of each fiber sample was extracted with 5 mL of 10% v/v of acetone in methanol. Next, the anti-oxidant activity was analysed as mentioned above.^31^

#### *In vivo* anti-aging activity by studying the oxidative stress in *C. elegans*

The maximum effectiveness of percent loading of the crude astaxanthin extract onto CA fibers was used to evaluate the oxidative stress effect in *C. elegans* 50 mg of the CA nanofibers containing the crude astaxanthin extract was extracted by 5 mL of ethanol. The evaporated crude extract was dissolved in 1% DMSO to afford feeding solutions at any concentration that will henceforth explain. *C. elegans* were handled according to the standard methods described previously.^39^ Large populations of *C. elegans* were cultured on solid Nematode Growth Media (NGM) agar plate and fed with *Escherichia coli* OP50 at 20 °C. Synchronized eggs were isolated from gravid adult worms by bleaching solution (12% NaClO and 10% 1 M NaOH) and then incubated at 20 °C overnight to obtain newly hatched L1 larvae. Oxidative stress in *C. elegans* was induced by exposure of 250 mM paraquat. Synchronized L1 larvae were allowed to develop and grow on agar plates containing OP50 mixed with only 1% (v/v) DMSO alone (control) or the crude extract solutions in 1% (v/v) DMSO. Fifty worms of *C. elegans* were transferred to 96-well plate containing S-basal medium mixed with OP50 for each concentration of the crude extract. For pre-treatment group, the crude astaxanthin extract-pretreated worms were transferred into the wells containing 1% DMSO or 250 mM paraquat. For co-treatment group, the 1% (v/v) DMSO-treated worms were transferred into the wells containing either the mixture of 1% (v/v) DMSO and 250 mM paraquat or the crude astaxanthin extract combined with 250 mM paraquat. For all-treatment conditions, the crude astaxanthin extract-pretreated worms were transferred into the wells containing the crude astaxanthin extract alone or crude astaxanthin extract combined with 250 mM paraquat. The worms were incubated at 20 °C for 12 h. The numbers of live and dead worms were counted and recorded every 2 h. The percent survival was then calculated.

#### Releasing study

In order to prepare the releasing media of astaxanthin, the releasing media contained 10% v/v of acetone and 0.5% w/v of SDS in PBS solution pH 7.4 to improve their solubility as stated in Taepaiboon *et al.*,^19^ with slightly modified conditions. The crude astaxanthin extract-loaded CA nanofibers were cut to afford a small piece of 50 mg and put in the 50 mL Erlenmeyer flask containing 20 mL of releasing media. The flask was covered by aluminium foil and paraffin film and put in a rotary shaker at 200 rpm. To investigate the amount of astaxanthin, the absorbance of the samples was measured at 475 nm at 5, 10, 15, 30, 60, 120, 240, 480, 720 and 1440 min, respectively. The amount of astaxanthins was calculated based on its calibration curve and reported as percentage cumulative release. The fresh media solution was filled at the same volume after sampling.

#### Stability of crude astaxanthin extract-loaded CA nanofibers

Freeze–thaw cycle method was used to evaluate the stability of astaxanthin derivatives in CA nanofibers compared with that of free crude astaxanthin extract according to Su *et al.*^40^ protocol with slight modifications. The accurate concentrations of astaxanthin solution were prepared with and without CA solution. After electrospinning, both of samples were transferred into an amber glass vial and incubated at −20 °C for 22 h. After that, frozen-samples were thawed at 50 °C for 2 h in an incubator. The freeze–thaw cycle process was repeated for 28 times. The stability of astaxanthins in the crude astaxanthin extract and crude astaxanthin extract loaded CA nanofibers was examined every 4 days using DPPH radical scavenging activity assay. Concisely, 50 mg of nanofibers were extracted with 5 mL of ethanol. The tyrosinase activity was performed using dopachrome method by Likhitwitayawuid *et al.*^41^ with some modifications. Mixtures of tyrosinase solution, l-dopa solution, ethanolic samples and phosphate buffer solution were measured for the absorbance at 473 nm using EnSpire Multimode Plate Reader (Perkin-Elmer, USA). The percentage tyrosinase inhibition was calculated as follows.
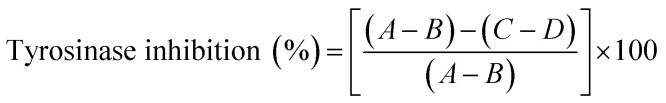
where *A* is the difference of absorbances (Abs_20 min_ − Abs_0 min_) of control, *B* is the difference of absorbances (Abs_20 min_ − Abs_0 min_) of blank of control, *C* is the difference of absorbances (Abs_20 min_ − Abs_0 min_) of sample, *D* is the difference of absorbances (Abs_20 min_ − Abs_0 min_) of blank of sample.

### Statistical analysis

Statistical analyses were carried out by Minitab Statistical Software (Minitab Version 16.2.4.0). All data were demonstrated as mean + SD values and compared to one-way analysis of variance (ANOVA). Tukey's test was used for a determination of the differences. The test with *P* < 0.05 was considered as a significant difference. All experiments were performed at least in triplicates.

## Conclusion

The optimal conditions for fabrication of CA electrospun nanofibers were studied. The effects of solvent systems and surfactant on fabrication of CA electrospun nanofibers play an important role in morphology of the fibers. The proper evaporation rate of the solvent systems and the appropriate amount of surfactant exhibited in a smaller average diameter size of fibers with uniform characteristic. The optimized conditions for fabrication of CA electrospun nanofibers was then used to produce crude astaxanthin extract-loaded CA nanofibers. It was found that the released free astaxanthins from crude astaxanthin extract-loaded CA nanofibers exhibited the capacity against the oxidative stress of *C. elegans* with significant values. The releasing profile of fibers in a modified buffer solution was that of a prolonged release. Moreover, the stability of released astaxanthins from the crude astaxanthin extract-loaded CA nanofibers was significantly higher compared to that of free astaxanthins in crude astaxanthin extract under an accelerated condition. The results suggest that drug loaded CA electrospun nanofibers are applicable for future drug delivery control applications.

## Conflicts of interest

There are no conflicts to declare.

## Supplementary Material

RA-008-C8RA08156E-s001
