# Supplementary material for: Electrospun cellulose acetate doped with astaxanthin derivatives from Haematococcus pluvialis for in vivo anti-aging activity
Source: RSC Adv. 2018 Nov 5;8(65):37151–8. doi: 10.1039/c8ra08156e (PMC9088889; doi:10.1039/c8ra08156e)
Supplement: RA-008-C8RA08156E-s001 [file RA-008-C8RA08156E-s001.pdf]

Electronic Supplementary Material (ESI) for RSC Advances.
This journal is © The Royal Society of Chemistry 2018

Supplementary data

Electrospun cellulose acetate doped with astaxanthin derivatives from *Haematococcus pluvialis* for in vivo anti-aging activity

Jukkrit Nootem[a], Pawanrat Chalorak[b], Krai Meemon[b], Withawat Mingvanish[a], Kornkanya Pratumyot[a], Leela Ruckthong[a], Choladda Srisuwannaket[a], Nakorn Niamnont[a,*]

[a]*Organic Synthesis, Electrochemistry & Natural Product Research Unit, Department of Chemistry, Faculty of Science, King Mongkut's University of Technology Thonburi, Bangkok, 10140, Thailand.*

[b]*Department of Anatomy, Faculty of Science, Mahidol University Bangkok, 10400, Thailand.*

Email: nakorn.nia@kmutt.ac.th

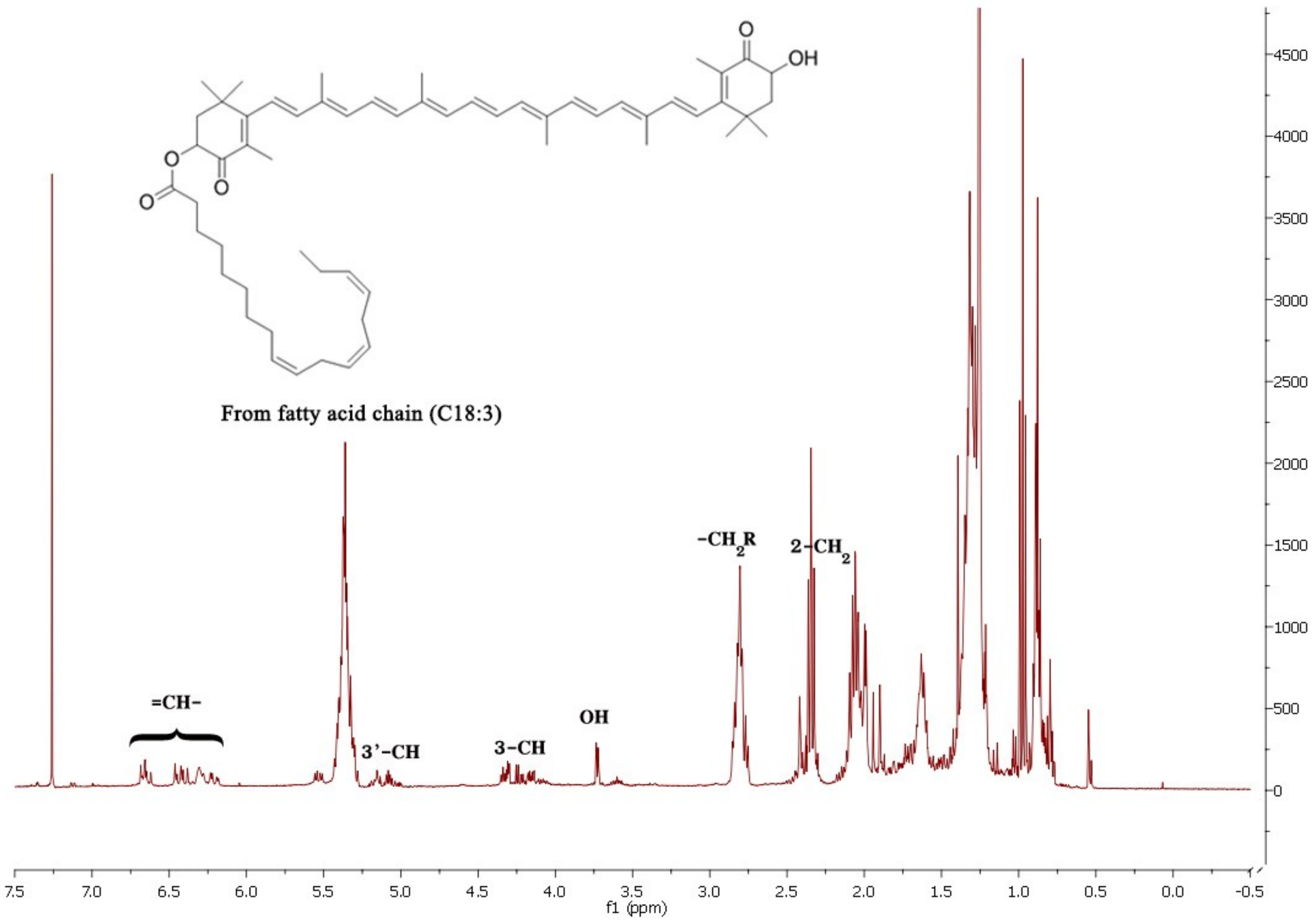

Figure. S1 ^{1}H-NMR 400 MHz spectra of astaxanthin monoester in $CDCl_3$.

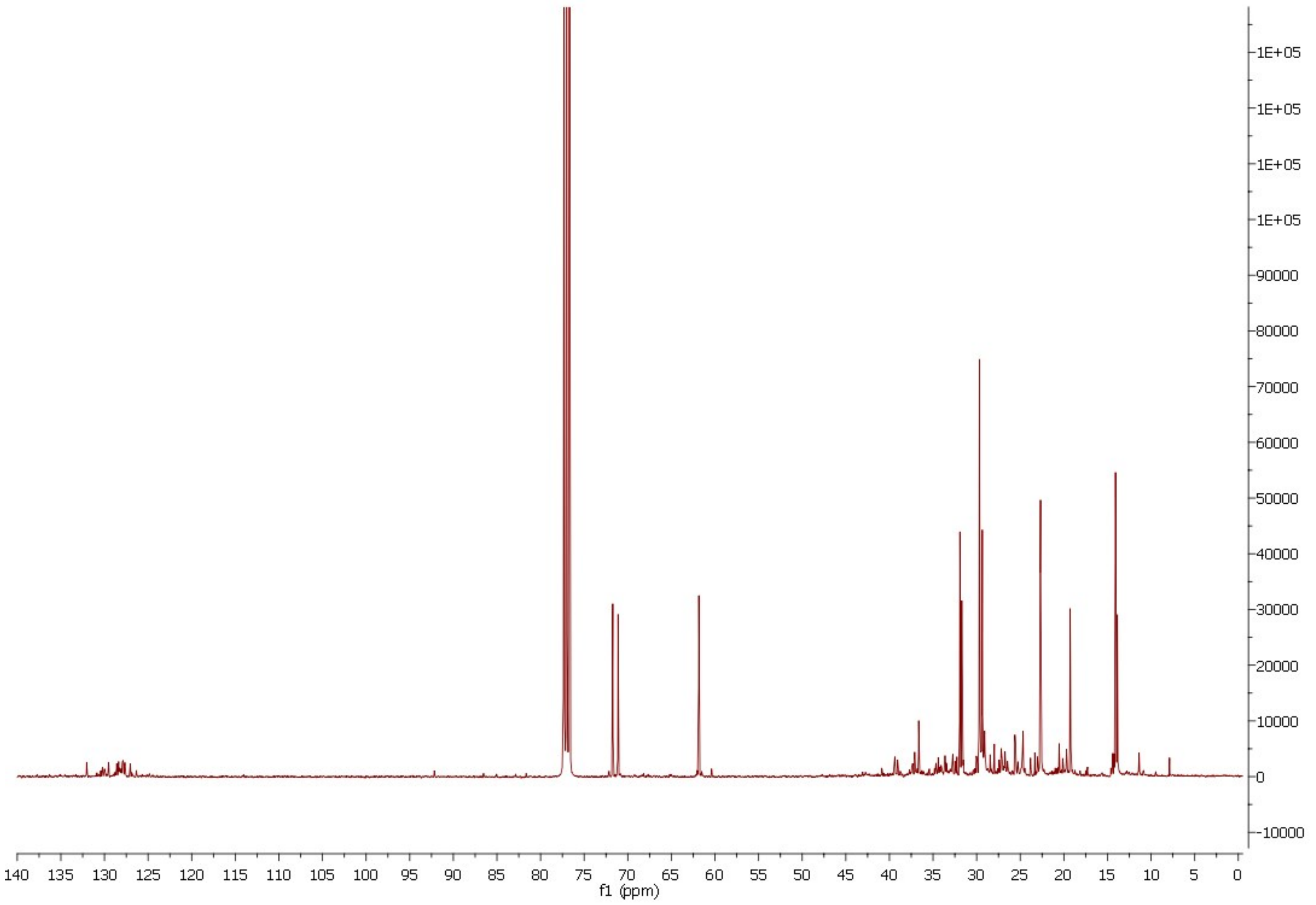

Figure. S2 ^{13}C-NMR 100 MHz spectra of astaxanthin monoester in $CDCl_3$.

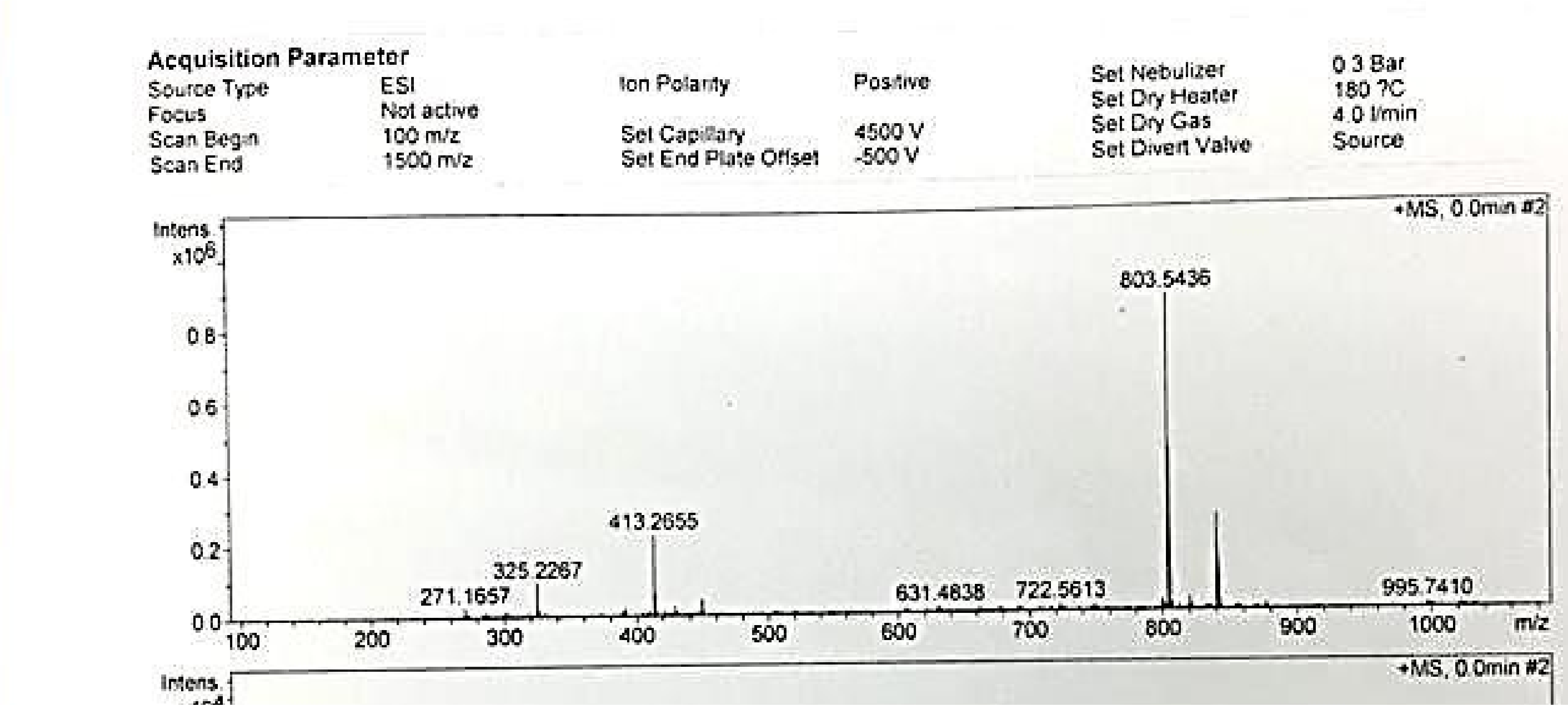

Figure. S3 HRMS spectra of astaxanthin monoester.

Table S1. Chlorophyll a, chlorophyll b and total carotenoids content of DPPH radical scavenging activity from *H. pluvialis* extraction. cE is means crude extraction.

Determination	Value (mg/g cE)
**Chlorophyll a**	8.72±0.25
**Chlorophyll b**	2.54±0.38
**Total carotenoids content**	10.75±0.16

Table S2. IR spectra of astaxanthin derivatives, CA fibers and astaxanthin derivatives loaded CA fibers.

Sample	Wave number (cm^{-1})	Vibration mode
**Astaxanthin derivatives**	3,485<br>2,964<br>1,652<br>978	O-H stretching<br>CH_2 stretching<br>C=C stretching<br>C-H stretching
**CA fibers**	3,472<br>2,925<br>1,741<br>1,368<br>1,228<br>1,037	O-H stretching<br>CH_2 stretching<br>C=C stretching<br>$C-CH_3$ stretching<br>C-O-C stretching<br>C-O-C stretching
**Astaxanthin derivatives loaded CA fibers**	3,489<br>2,932<br>1,750<br>1,660<br>1,365<br>1,231<br>1,309	O-H stretching<br>CH_2 stretching<br>C=O stretching<br>$C-CH_3$ stretching<br>$C-CH_3$ stretching<br>C-O-C stretching<br>C-O-C stretching
